# Downregulation of CD47 and CD200 in patients with focal cortical dysplasia type IIb and tuberous sclerosis complex

**DOI:** 10.1186/s12974-016-0546-2

**Published:** 2016-04-19

**Authors:** Fei-Ji Sun, Chun-Qing Zhang, Xin Chen, Yu-Jia Wei, Song Li, Shi-Yong Liu, Zhen-le Zang, Jiao-Jiang He, Wei Guo, Hui Yang

**Affiliations:** Department of Neurosurgery, Xinqiao Hospital, Third Military Medical University, 2-V Xinqiao Street, Chongqing, 400037 China; Department of Neurosurgery, Lanzhou General Hospital of Chinese People’s Liberation Army, Lanzhou, China; Department of Neurosurgery, Tangdu Hospital, Fourth Military Medical University, Xian, China

**Keywords:** Focal cortical dysplasia type IIb, Tuberous sclerosis complex, Inflammation, CD47, Signal regulatory protein α, CD200, CD200 receptor

## Abstract

**Background:**

Focal cortical dysplasia type IIb (FCD IIb) and tuberous sclerosis complex (TSC) are well-recognized causes of chronic intractable epilepsy in children. Accumulating evidence suggests that activation of the microglia/macrophage and concomitant inflammatory response in FCD IIb and TSC may contribute to the initiation and recurrence of seizures. The membrane glycoproteins CD47 and CD200, which are highly expressed in neurons and other cells, mediate inhibitory signals through their receptors, signal regulatory protein α (SIRP-α) and CD200R, respectively, in microglia/macrophages. We investigate the levels and expression pattern of CD47/SIRP-α and CD200/CD200R in surgically resected brain tissues from patients with FCD IIb and TSC, and the potential effect of soluble human CD47 Fc and CD200 Fc on the inhibition of several proinflammatory cytokines associated with FCD IIb and TSC in living epileptogenic brain slices in vitro. The level of interleukin-4 (IL-4), a modulator of CD200, was also investigated.

**Methods:**

Twelve FCD IIb (range 1.8–9.5 years), 13 TSC (range 1.5–10 years) patients, and 6 control cases (range 1.5–11 years) were enrolled. The levels of CD47/SIRP-α and CD200/CD200R were assessed by quantitative real-time polymerase chain reaction and western blot. The expression pattern of CD47/SIRP-α and CD200/CD200R was investigated by immunohistochemical analysis, and the cytokine concentrations were measured by enzyme-linked immune-sorbent assays.

**Results:**

Both the messenger RNA and protein levels of CD47, SIRP-α, and CD200, as well as the mRNA level of IL-4, were downregulated in epileptogenic lesions of FCD IIb and TSC compared with the control specimens, whereas CD200R levels were not significantly changed. CD47, SIRP-α, and CD200 were decreasingly expressed in dysmorphic neuron, balloon cells, and giant cells. CD47 Fc and CD200 Fc could inhibit IL-6 release but did not suppress IL-1β or IL-17 production.

**Conclusions:**

Our results suggest that microglial activation may be partially caused by CD47/SIRP-α- and CD200/CD200R-mediated reductions in the immune inhibitory pathways within FCD IIb and TSC cortical lesions where chronic neuroinflammation has been established. Upregulation or activation of CD47/SIRP-α and CD200/CD200R may have therapeutic potential for controlling neuroinflammation in human FCD IIb and TSC.

**Electronic supplementary material:**

The online version of this article (doi:10.1186/s12974-016-0546-2) contains supplementary material, which is available to authorized users.

## Background

Malformations of cortical development (MCD) are major causes of intractable pediatric epilepsy [1]. Focal cortical dysplasia (FCD) and tuberous sclerosis complex (TSC) are two common classes of MCD [2]. FCD is characterized by sporadic architectural and cytoarchitectural malformations of the cerebral cortex and has been currently classified into type I, type II, type III, and additional subtypes. FCD type II is composed of type IIa (cortical dyslamination and dysmorphic neurons) and type IIb (cortical dyslamination, dysmorphic neurons, and balloon cells) [3]. TSC is an autosomal dominant disorder caused by mutations in the TSC1 or TSC2 genes and is associated with lesions in multiple organ systems, including cortical tubers in the brain [4]. In addition to having progressive and recurrent seizures in common, TSC cortical tubers also have a number of histopathological features similar to focal cortical dysplasia type IIb (FCD IIb), such as disorganized lamination, dysmorphic neurons, and giant cells, suggesting common mechanisms responsible for structural abnormalities and epileptogenesis [5]. Additionally, mammalian target of rapamycin complex 1 activation and autophagy defect are observed in both the TSC giant cells and FCD IIb balloon cells [6, 7]. And both TSC and FCD IIb cortical lesions express abnormally phosphorylated tau protein, an important microtubule-associated protein that in aging adults produces dementia but in immature brain interferes with cellular lineage, neuroblast polarity and migration, and especially cellular growth and morphogenesis, features they share with hemimegalencephaly [8, 9]. Although many studies have indicated that seizures are likely to originate within the dysplastic cortical lesions in FCD IIb and the cortical tubers in TSC [5], the cellular and molecular mechanisms underlying the epileptogenesis of FCD IIb and TSC are still unknown.

Evidence from experimental and clinical studies suggests that activation of immune systems occurs in various focal epilepsies and that the inflammatory response may contribute to the generation and progression of seizures [10]. Activation of microglia/macrophages, associated with prominent and sustained overexpression of proinflammatory mediators, has been described in a variety of epileptogenic specimens, including FCD IIb and TSC [11]. As the major immune cells in the central nervous system (CNS), microglia/macrophages behavior is tightly regulated by the integration of activating and inhibitory signals [12]. To date, most studies have focused on the role of the microglial/macrophage activating pathways and the proinflammatory mediators released by activated microglia in MCD [13–17]; little attention has been paid to the expression pattern and potential effect of microglial/macrophage inhibitory factors, such as CD47 and CD200. CD47 or integrin-associated protein is an ubiquitously expressed cell surface glycoprotein that was originally identified in association with the integrin α_v_β_3_ [18]. In the CNS, CD47 is distributed on neurons as well as on other types of cells, serving as a ligand for signal regulatory protein α (SIRP-α), an immune inhibitory receptor on microglia and neurons [19–22]. The interaction between CD47 and SIRP-α results in the inhibition of microglial/macrophage phagocytosis [19, 23], and ligation of SIRP-α by CD47-Fc fusion proteins was found to prevent the phenotypic and functional maturation of immature dendritic cells (DCs) and to inhibit cytokine production by mature DCs [24]. Another immune inhibitory molecular is CD200 which is a surface molecule belonging to the immunoglobulin supergene family [25]. Similar to CD47, CD200 is widely expressed in several cells in the CNS, including neurons, while its receptor (CD200R) is primarily present on microglia in the brain [26, 27]. Mice lacking CD200 display spontaneous microglial activation and have a more rapid onset of experimental autoimmune encephalomyelitis (EAE), an animal model of multiple sclerosis (MS) [28]. Similarly, in vitro blockade of CD200R on macrophages leads to elevated release of IL6 and neuronal cell death in co-cultures with hippocampal neurons expressing CD200 [29].

In the present study, we investigated the levels and expression pattern of CD47, SIRP-α, CD200, and CD200R in surgically resected brain tissues from patients with FCD IIb and TSC. To assess the potential roles of CD47 and CD200 on the epileptogenesis of FCD IIb and TSC, we examined the concentrations of several proinflammatory cytokines (IL1-β, IL-6, and IL-17), which are associated with the epileptogenesis of FCD IIb and TSC [15–17, 30], in living epileptogenic brain slices treated with soluble recombinant human CD47 Fc chimera protein or CD200 Fc chimera protein compared with the vehicle-treated controls. We also evaluated the level of IL-4, which has been shown to increase the expression of CD200 [31, 32].

## Methods

### Subjects

A total of 25 subjects were enrolled: 12 FCD IIb and 13 TSC patients. The cases included in this study were obtained from the Department of Neurosurgery of the Xinqiao Hospital (Third Military Medical University, Chongqing, China). Informed consent and written permission for all procedures were obtained before surgery from the patients or their direct relatives. The clinical characteristics of the patients were summarized in Table 1. All procedures and experiments were conducted under the guidelines approved by the Ethics Committee of the Third Military Medical University, and all investigations were performed in accordance with the criteria of the Declaration of Helsinki of the World Medical Association.

**Table 1:** Clinical features of patients with FCD IIb and TSC

Case no.	Gender	Age at surgery	Diagnosis	Seizure focus	Epilepsy duration (year)	Seizure frequency (per month)	Engel’s class	Application in the present study
1	F	7	FCD IIb	F	5	45	I	qPCR, WB, IHC,
2	F	1.8	FCD IIb	F	1.2	105	II	ELISA
3	M	3.5	FCD IIb	T	3	65	I	qPCR, WB, IHC,
4	F	7.5	FCD IIb	F	6.5	30	I	qPCR, WB, IHC,
5	M	5	FCD IIb	T	4	80	II	ELISA
6	M	4.3	FCD IIb	O	3.5	120	I	qPCR, WB, IHC,
7	F	9.5	FCD IIb	P	8	25	I	ELISIA
8	F	5.5	FCD IIb	F	4	60	I	qPCR, WB, IHC,
9	M	6	FCD IIb	P	4.5	35	III	qPCR, WB, IHC,
10	F	2	FCD IIb	T	1	55	I	qPCR, WB, IHC,
11	M	2.3	FCD IIb	O	1.3	70	I	qPCR, WB, IHC,
12	F	7	FCD IIb	T	5.5	30	III	ELISA
13	F	6	TSC	P	4.8	60	I	qPCR, WB, IHC,
14	M	2.1	TSC	P	1.2	110	I	qPCR, WB, IHC,
15	M	3.9	TSC	T	3	82	I	qPCR, WB, IHC,
16	F	7	TSC	F	5.6	26	II	ELISA
17	F	5.5	TSC	T	4	48	I	ELISA
18	F	4.8	TSC	P	3.8	75	I	qPCR, WB, IHC,
19	M	10	TSC	T	8.1	30	I	qPCR, WB, IHC,
20	F	6	TSC	F	4.5	25	III	ELISIA
21	M	5.5	TSC	F	4	31	I	qPCR, WB, IHC,
22	M	3	TSC	T	2.3	55	I	qPCR, WB, IHC,
23	M	1.5	TSC	O	0.9	100	III	qPCR, WB, IHC,
24	F	4	TSC	P	2.9	50	I	qPCR, WB, IHC,
25	F	3.5	TSC	F	2.6	45	II	ELISA

*F* female, *M* male, *FCD IIb* focal cortical dysplasia type IIb, *TSC* tuberous sclerosis complex, *F* frontal lobe, *O* occipital lobe, *P* parietal lobe, *T* temporal lobe, *ELISA* enzyme-linked immune sorbent assay, *qPCR* quantitative real-time polymerase chain reaction, *WB* western blot, *IHC* immunohistochemistry (including double-labeled immunofluorescence)

All epileptogenic tissue samples were obtained from regions identified as dysplastic by magnetic resonance imaging and confirmed post hoc by neuropathology. For the FCD specimens, we followed the current classification system of the International League Against Epilepsy (ILAE) for grading the degree of FCD [3], and only patients with FCD IIb were included. All diagnoses of TSC fulfilled the diagnostic criteria for TSC [4]. Furthermore, clinical mutation analyses of the TSC1 and TSC2 loci were performed by denaturing high-performance liquid chromatography (DHPLC) to confirm our diagnoses.

For the control experiments, the histologically normal cortex tissues were obtained at autopsy from six control patients (male/female 3/3; mean age 4.83; range 1.5–11 years), who did not have a history of seizures or other neurological diseases. All the autopsies were performed within 6 h after death. Within this post-mortem interval, it is well documented that most proteins are stable and therefore well preserved [33]. Two neuropathologists also helped to review the autopsy cases, and both gross and microscopic examinations revealed no structural abnormality.

### Tissue processing

Resected brain tissues were immediately divided into two parts. One portion was immediately placed in a cryovial that had been soaked in buffered diethylpyrocarbonate (1:1000) for 24 h and was then snap-frozen in liquid nitrogen. Frozen samples were maintained at −80 °C until they were used for quantitative real-time polymerase chain reaction (qPCR) and western blot analysis. The second portion of the brain tissue was fixed in 10 % buffered formalin for 48 h and embedded in paraffin, sectioned at 6 μm for immunohistochemistry (IHC) or 10 μm for double-labeled immunofluorescence, and mounted on polylysine-coated slides.

### Brain slice preparation

As described previously [34, 35], brain tissue specimens were resected and immediately submerged in ice-cooled (0–4 °C), oxygenated (95 % O_2_ and 5 % CO_2_) cutting solution containing (in mM) 210 sucrose, 2.5 KCl, 1.02 NaH_2_PO_4_, 0.5 CaCl_2_, 10 MgSO_4_, 26.19 NaHCO_3_, and 10 D-glucose, pH 7.4. The specimens were then transferred rapidly (within 5–10 min) to our laboratory, dissected into appropriate blocks, and cut into 300 μm thick using a vibratome (LEICA VT1000S, Leica Microsystem Inc., Bannockburn). The slices were then incubated with soluble recombinant human CD47 Fc chimera protein (Catalogue Number: 4670-CD, R&D Systems) or CD200 Fc chimera protein (Catalogue Number: 2724-CD, R&D Systems) in a concentration of 5 μg/ml in oxygenated (95 % O_2_ and 5 % CO_2_) artificial cerebrospinal fluid (ACSF) containing the following (in mM): NaCl, 124; KCl, 5; NaH_2_PO_4_, 1.25; MgSO_4_, 1.2; NaHCO_3_, 26; CaCl_2_, 2; and glucose, 10 (pH 7.4), as CD47 Fc and CD200 Fc in this dose suppress the production of several inflammatory cytokines in human DCs and activated microglia, respectively [24, 36]. The control slices were incubated with sterile ACSF which was used for the dilutions of CD47 Fc and CD200 Fc [37]. All the slices were maintained at 37 °C for subsequent cytokine analysis by enzyme-linked immune-sorbent assays (ELISAs).

### Quantitative real-time polymerase chain reaction

Total RNA was isolated from each sample using a TRIzol reagent isolation kit (Invitrogen, La Jolla, CA), according to the manufacturer’s instructions. The concentration and purity of RNA were determined spectrophotometrically at 260/280 nm with a nanodrop spectrophotometer (Ocean Optics, Dunedin, FL). One microgram of total RNA was reverse-transcribed into single-stranded complementary DNA with oligo dT primer (TakaRa, Otsu, Japan). PCR primers were designed based on the complementary DNA sequence and synthesized by TaKaRa Biotechnology Company (Chongqing, China). The primers used were as follows: CD47 (forward: GAAGATGGATAAGAGTGATGCTGTC; reverse: ACCTGGGACGAAAAGAATGG), SIRP-α (forward: GGCTCCTGGTGAATGTATCTGC; reverse: GTGTTCTCAGCGGCGGTATT), CD200 (forward: GTCTACCTACAGCCTGGTTTGG; reverse: GCTGGGTAATGTTTATCTTGTCCTT), CD200R (forward: ACTAAGCAAGAATACTGGAGCAATG; reverse: TCAACAACCAAATGAATCCCAC), and IL-4 (forward: GCTACCCTGTTCGGCTTTCCT; reverse: TCCCGTGGTTGTCCTTGTGT). The amplification conditions was as follows: 95 °C for 5 min (1 cycle), followed by 40 cycles of 95 °C for 30 s, 60 °C for 30 s. The relative quantification of each product versus the reference gene β-actin was evaluated by the 2^−△△ct^ method.

### Western blot

Equal amounts of protein (60 μg/lane) were separated by sodium dodecyl sulfate-polyacrylamide gel electrophoresis analysis on 10 % gels. The separated proteins were transferred onto polyvinylidene fluoride membranes (Millipore, Temecula, CA, USA) using a semidry electroblotting system (Transblot SD; Bio-Rad). The blots were incubated overnight at 4 °C in Tris-buffered saline with Tween (TBST, 20 mmol/l Tris–HCl, pH 8.0, 150 mmol/l NaCl, 0.5 % Tween-20) with 5 % nonfat dry milk containing the following primary antibodies: anti-glyceraldehyde-3-phosphate dehydrogenase (GAPDH, rabbit monoclonal, Cell Signaling Technology, Beverly, MA, USA; 1:2000), anti-CD47 (rabbit polyclonal; GeneTex, Inc., San Antonio, TX, USA; 1:500), anti-SIRP-α (rabbit polyclonal; GeneTex, Inc., San Antonio, TX, USA; 1:1000), anti-CD200 (rabbit polyclonal; Santa Cruz Biotechnology, Santa Cruz, CA; 1:500), and anti-CD200R (goat polyclonal; Santa Cruz Biotechnology, Santa Cruz, CA; 1:500). After several washes in TBST, the samples were incubated with a horseradish peroxidase-conjugated goat anti-rabbit secondary antibody (Santa Cruz Biotechnology, Santa Cruz, CA; 1:2000) or donkey anti-goat secondary antibody (Santa Cruz Biotechnology, Santa Cruz, CA; 1:2000) for 1 h at room temperature. The antibodies were visualized using enhanced chemiluminescence. Immunoreactive bands were analyzed densitometrically and normalized to GAPDH using a Gel-Pro analyzer.

### Immunohistochemistry and double-labeled immunofluorescence

Six-micrometer-thick paraffin-embedded sections were mounted on polylysine-coated slides and used for IHC. The paraffin-embedded sections were de-paraffinized, rehydrated, and incubated for 30 min in 0.3 % H_2_O_2_ diluted in methanol to quench endogenous peroxidase activity. All of the samples were placed into phosphate buffered saline (0.01 M, pH 7.3) and heated in a microwave oven for antigen retrieval. The sections were then incubated for 1 h at room temperature followed by incubation with the following primary antibodies overnight at 4 °C: anti-CD47 (rabbit polyclonal; GeneTex, Inc., San Antonio, TX, USA; 1:100), anti-SIRP-α (rabbit polyclonal; GeneTex, Inc., San Antonio, TX, USA; 1:100), anti-CD200 (rabbit polyclonal; Abcam, Cambridge, UK; 1:100), and anti-CD200R (rabbit polyclonal; ab198010, Abcam, Cambridge, UK; 1:100). After three rinses, the sections were incubated with the secondary goat anti-rabbit immunoglobulin conjugated to peroxidase-labeled dextran polymer (Envision + System-HRP; Boster, Wuhan, China) for 1 h at 37 °C. The immunoreactions were visualized using 3,3-diaminobenzidine (DAB; Boster, Wuhan, China). The sections were counterstained with hematoxylin, dehydrated, and coverslipped. No immunoreactive cells were detected in negative control experiments, which included application of the secondary antibody alone, preabsorption with a tenfold excess of a specific blocking antigen or incubation with an isotype-matched rabbit polyclonal antibody. Immunoreactivity (IR) was observed under a Leica DMIRB microscope (Leica, Nussloch, Germany).

For double-labeled immunofluorescence, sections were incubated overnight at 4 °C with the following primary antibodies: anti-CD47 (rabbit polyclonal; GeneTex, Inc., San Antonio, TX, USA; 1:100), anti-SIRP-α (rabbit polyclonal; GeneTex, Inc., San Antonio, TX, USA; 1:100), anti-CD200 (rabbit polyclonal; Abcam, Cambridge, UK; 1:100), and anti-CD200R (rabbit polyclonal; Abcam, Cambridge, UK; 1:100), combined with anti-neurofilament (NF; mouse monoclonal, Boster, Wuhan, China; 1:200), anti-glial fibrillary acidic protein (GFAP; mouse monoclonal, Sigma; 1:500), and anti-human leukocyte antigen-DR (HLA-DR; mouse monoclonal, Dako, Denmark; 1:100). After three washes, the sections were incubated with a mixture of Alexa Fluor 488-conjugated goat anti-rabbit IgG (Invitrogen; 1:500) and Alexa Fluor 594-conjugated goat anti-mouse IgG (Invitrogen; 1:500) for 1 h at 37 °C. 4’,6-diamidino-2-phenylindole (DAPI, 10 μg/ml, Beyotime, Nanjing, China) was used to counterstain the cell nuclei. The fluorescent sections were visualized and photographed with a laser scanning confocal microscope (TCS-TIV; Leica, Nussloch, Germany).

### Evaluation of immunoreactivity and cell counting

The evaluation of specific immunoreactivity, the presence or absence of various histopathological parameters, and cell counting were assessed by two independent observers blind to clinical data. The overall concordance was >90 %, and the overall kappa value ranged from 0.87 to 0.98. When a disagreement occurred, independent reevaluation was performed by both observers to define the final score. A semi-quantitative analysis was performed as previously described [38, 39]. Using a Leica DMIRB microscope to examine a total microscopic area of 781.250 μm^2^ (200 high-power non-overlapping fields of 0.0625 × 0.0625 mm width, using a square grid inserted into the eyepiece) in each section. The staining intensity was evaluated using a semi-quantitative three-point scale where the IR was defined as follows: − absent (0), + weak (1), ++ moderate (2), and +++ strong staining (3). These scores represent the predominant staining intensity in each section and were calculated as the average of the selected fields. In addition, we assessed the number of positive cells within the cortical lesions of FCD and TSC to obtain the relative proportion of immunoreactive cells. This frequency score was evaluated by three distinct categories: (1) single to 10 %; (2) 11–50 %; and (3) >50 %. The product of these two values (intensity and frequency scores) was used to obtain the total score as previously reported [13, 40].

To analyze the correlation between expression levels (IR scores) of CD47, SIRP-α, and CD200 in FCD IIb and TSC specimens and the number of activated microglia labeled by HLA-DR [41], quantitative analysis was performed as previously described [13, 42]. Images of two representative fields per section (magnification 60 times) were captured and digitized with a laser scanning confocal microscope (TCS-TIV; Leica, Nussloch, Germany). The number of HLA-DR-positive cells was counted.

### Cytokine ELISAs

Supernatants of the living cortical brain slices were collected for ELISAs after 12 h of incubation, since the viability of the slices in ACSF is still good at 12 h after resection [43]. The concentrations of IL-1β (Bender MedSystems, Vienna, Austria), IL-6, and IL-17 (PeproTech, Rocky Hill, NJ, USA) were measured according to the manufacturer’s instructions. ELISAs were performed in duplicate, and the values were calculated from a standard curve generated for each result. The data were expressed in picogram per milliliter.

### Statistical analyses

The data are expressed as the mean ± SD, and analysis was performed using SPSS 19.0 package (SPSS Inc., Chicago, IL, USA). Differences were assessed by the chi-square test for gender and IR scores. And differences in age, epilepsy duration, seizure frequency, mRNA levels, and protein levels were assessed by the Kruskal–Wallis test and Bonferroni correction. Mann–Whitney *U* test was used for the comparison of cytokine concentrations. Spearman’s rank correlation test was used for bivariate correlation analyses. *P* < 0.05 was considered significant.

## Results

### Clinical variables and histological features

The clinical variables of all the subjects who were enrolled in this study were summarized in Table 2. There were no significant differences in gender and age between the control subjects and epilepsy patients (FCD IIb and TSC) (*P* > 0.05). Moreover, no significant differences in gender, age, epilepsy duration, and seizure frequency were also found between FCD IIb and TSC (*P* > 0.05).

**Table 2:** Clinical variables of the subjects in epilepsy groups and autopsy controls

Variable	Control	FCD IIb	TSC	*P* value
Number	6	12	13	
Gender ratio (M/F)	3/3	5/7	6/7	*P > 0.05* ^a^
Age	4.83 ± 3.47	5.12 ± 2.43	4.85 ± 2.24	*P > 0.05* ^b^
Range	1.5–11	1.8–9.5	1.5–10	
Epilepsy duration (year)	NA	3.96 ± 2.16	3.76 ± 1.96	*P > 0.05* ^b^
Range	NA	1–8	0.9–8.1	
Seizure frequency (per month)	NA	60.00 ± 30.23	57.67 ± 28.82	*P > 0.05* ^b^
Range	NA	25-120	25–110	

*F* female, *M* male, *NA* not applicable. Mean ± SD, *P* values calculated by ^a^chi-square test, ^b^Kruskal–Wallis test, Bonferroni correction

The FCD subjects included displayed the histopathological features of FCD IIb according to the criteria of the ILAE, including the presence of dysmorphic neurons and balloon cells (Additional file 1: Figure S1B). As previously described, the histopathological features of the TSC cortical tubers include cortical dyslamination, dysmorphic neurons, and giant cells (Additional file 1: Figure S1C). Dysmorphic neurons displayed large nucleoli, abnormal soma size and orientation, and abundant Nissl substance staining. Balloon cells (giant cells in TSC) were defined as morphologically abnormal cells with a thin membrane, eosinophilic cytoplasm, and one or more eccentrically located nuclei.

### Quantitative real-time PCR analysis of CD47, SIRP-α, CD200, and CD200R

**Fig. 1:**
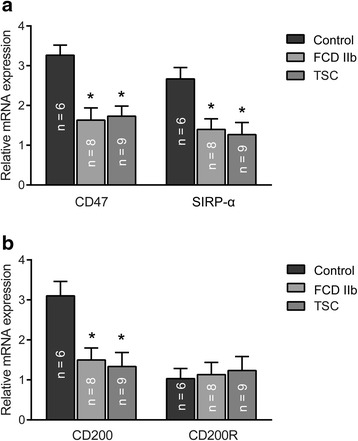
Messenger RNA expression of CD47, SIRP-α, CD200, and CD200R in control, FCD IIb, and TSC specimens. Quantitative real-time PCR analysis showed that the mRNA levels of CD47 (**a**) and its receptor SIRP-α (**a**) and CD200 (**b**) were significantly decreased in FCD IIb and TSC compared with control specimens, while CD200R (**b**) mRNA level was not significantly changed. There were no significant differences in CD47, CD200, and their receptors between FCD IIb and TSC specimens. The *bar charts* represent mean ± SD, *P < 0.05, FCD IIb, TSC versus controls, Kruskal–Wallis test, and Bonferroni correction

Quantitative real-time PCR (qPCR) was employed to qualify the messenger RNA (mRNA) levels of CD47, SIRP-α, CD200, and CD200R in the total homogenates from FCD IIb cortical lesions, TSC cortical tubers, and control tissues, with β-actin as an internal control. Decreased mRNA expression of CD47, SIRP-α, and CD200 were observed in both the FCD IIb and TSC lesions compared with control tissues (*P* < 0.05; Fig. 1a, b), whereas the CD200R mRNA level was not significantly changed (*P* > 0.05; Fig. 1b).

### Western blot analysis of CD47, SIRP-α, CD200, and CD200R

**Fig. 2:**
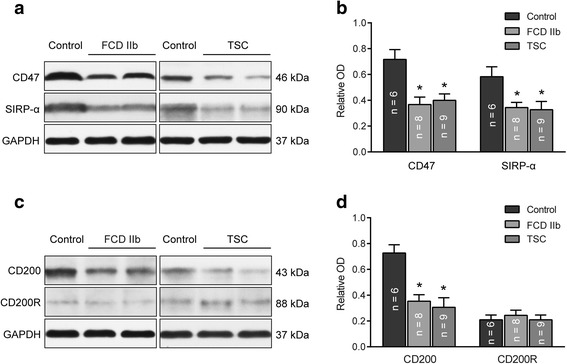
Protein expression of CD47, SIRP-α, CD200, and CD200R in control, FCD IIb, and TSC specimens. **a**, **c** Representative immunoblot of CD47, CD200 and their receptors in control, FCDIIb and TSC samples. Expression of GAPDH (as reference protein) is shown in the same protein extracts. Densitometric analysis of western blot showed that CD47 (**b**), SIRP-α (**b**), and CD200 (**d**) protein levels were significantly decreased in FCD IIb and TSC compared with the control specimens, while CD200R (**d**) protein level was not significantly changed. There were no significant differences in CD47, SIRP-α, CD200, and CD200R between FCD IIb and TSC specimens. The *bar charts* represent mean ± SD, *P < 0.05, FCD IIb, TSC versus controls, Kruskal–Wallis test and Bonferroni correction

We used western blot to examine the protein levels of CD47, SIRP-α, CD200, and CD200R in the total homogenates from FCD IIb cortical lesions, TSC cortical tubers, and control tissues, with GAPDH as an internal control. Similar to that in mRNA expression, the protein levels of CD47, SIRP-α, and CD200 were downregulated in both the FCD IIb and TSC lesions compared with control tissues (*P* < 0.05; Fig. 2a–d), while the CD200R protein level was not significantly altered (*P* > 0.05; Fig. 2c, d).

### Immunohistochemistry analysis of CD47, SIRP-α, CD200, and CD200R

#### CD47

**Fig. 3:**
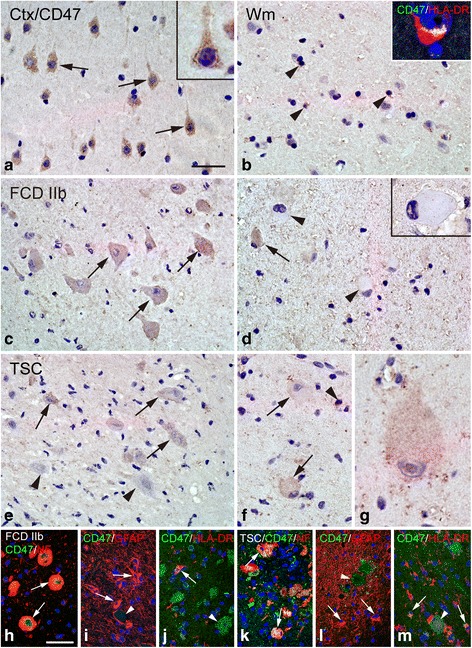
CD47 IR in control, FCD IIb, and TSC specimens. **a**, **b** CD47 IR in control specimens. **a** Strong somatic staining of CD47 in neurons (*arrows* and *inset*) within cortex. **b** Sporadic CD47 IR in glial cells (*arrowheads*) within white matter and co-localization of CD47 (*green*) and HLA-DR (*red*) in a microglia (*inset*). **c**, **d** CD47 IR in cortical lesions of FCD IIb. **c** Weak CD47 IR in dysmorphic neurons (*arrows*). **d** Negative balloon cells (*arrowheads* and *inset*) and weak CD47 IR in balloon cells (*arrows*). **e**–**g** CD47 IR in cortical tubers of TSC. **e** Negative dysmorphic neurons (*arrowheads*) and weak to moderate staining in dysmorphic neurons (*arrows*). **f** Weak CD47 IR in giant cells (*arrows*) and in a glial cell (*arrowhead*). **g** High magnification showing a giant cell with weak CD47 IR. **h**–**j** Double labeling in cortical lesions of FCDIIb specimens. **h** Co-localization of CD47 (*green*) with NF (*red*) in dysmorphic neurons. **i** Absence of co-localization between CD47 (*green*) and GFAP (*red*) in astrocytes (*arrows*: astrocytes, *arrowhead*: dysmorphic neuron). **j** Co-localization of CD47 (*green*) and HLA-DR (*red*) in a microglia (*arrow*; *arrowhead*: dysmorphic neuron). **k**–**m** Double labeling in cortical tubers of TSC specimens. **k** Co-localization of CD47 (*green*) with NF (*red*) in giant cells. **l** Absence of co-localization between CD47 (*green*) and GFAP (*red*) in astrocytes (*arrows*: astrocytes, *arrowhead*: giant cell). **m** Co-localization of CD47 (*green*) and HLA-DR (*red*) in microglia (*arrows*; *arrowhead*: giant cell). *Scale bars*: **a** 40 μm; **b** 35 μm; **d**, **e**: 50 μm; **f** 30 μm, **g** 20 μm; **h**–**m** 50 μm

**Fig. 4:**
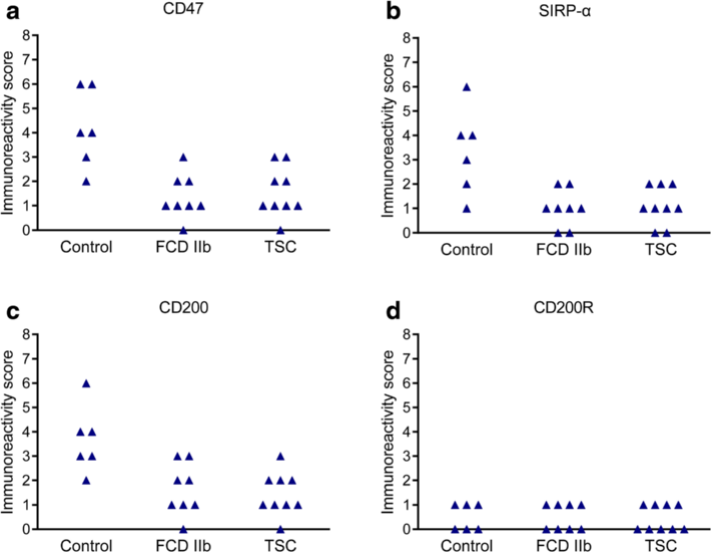
Semi-quantitative evaluation of CD47, SIRP-α, CD200, and CD200R IR scores (total score; for details refer to “Methods” section) in control, FCDIIb, and TSC specimens. IR scores of CD47 (**a**), SIRP-α (**b**), and CD200 (**c**) in FCD IIb and TSC specimens were decreased compared with those in controls, *P* < 0.05, chi-square test; whereas IR score of CD200R (**d**) was not altered, *P* > 0.05, chi-square test. There were no significant differences in IR scores of CD47, SIRP-α, CD200, and CD200R between FCD IIb and TSC specimens. *P* > 0.05, chi-square test

**Fig. 5:**
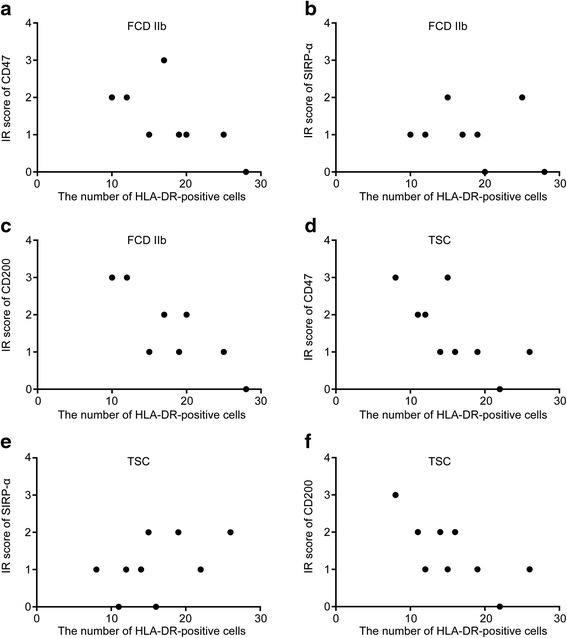
Correlation between IR scores (relative expression levels) of CD47, SIRP-α, CD200, and the number of HLA-DR-positive cells (activated microglia). **a** Scatter plot showing the significant negative correlation between the IR score of CD47 and the number of HLA-DR-positive cells in FCD IIb (*P* < 0.05, *r* = −0.74). **b** No significant correlation between the IR score of SIRP-α and the number of HLA-DR-positive cells in FCD IIb (*P* > 0.05, *r* = −0.31). **c** Significant negative correlation between the IR score of CD200 and the number of HLA-DR-positive cells in FCD IIb (*P* < 0.05, *r* = −0.78). **d** Scatter plot showing the significant negative correlation between the IR score of CD47 and the number of HLA-DR-positive cells in TSC (*P* < 0.05, *r* = −0.75). **e** No significant correlation between the IR score of SIRP-α and the number of HLA-DR-positive cells in TSC (*P* > 0.05, *r* = 0.48). **f** Significant negative correlation between the IR score of CD200 and the number of HLA-DR-positive cells in FCD IIb (*P* < 0.05, *r* = −0.71). Correlation analysis performed with Spearman’s rank correlation coefficient

Histologically normal autopsy cortex displayed strong somatic CD47 IR in pyramidal neurons (Fig. 3a) and showed moderate and sporadic CD47 IR in glial cells within white matter (Fig. 3b). In FCD IIb and TSC specimens, weak or undetectable CD47 IR was observed in dysmorphic neurons (Fig. 3c, e). Weak or undetectable CD47 IR was also detected in balloon cells of FCD IIb and in giant cells of TSC (Fig. 3d, f, and g). The CD47 IR score was significantly decreased in both the FCD IIb and TSC specimens compared with the controls (*P* < 0.05; Fig. 4a) and showed significant negative correlation with the number of HLA-DR-positive cells in FCD IIb (Fig. 5a) and TSC (Fig. 5d). Double labeling demonstrated the co-localization of CD47 IR with NF in certain dysmorphic neurons and giant cells (Fig. 3h, k) and with HLA-DR in certain microglia (Fig. 3j, m). GFAP-positive astrocytes did not display CD47 IR (Fig. 3i, l).

### SIRP-α

**Fig. 6:**
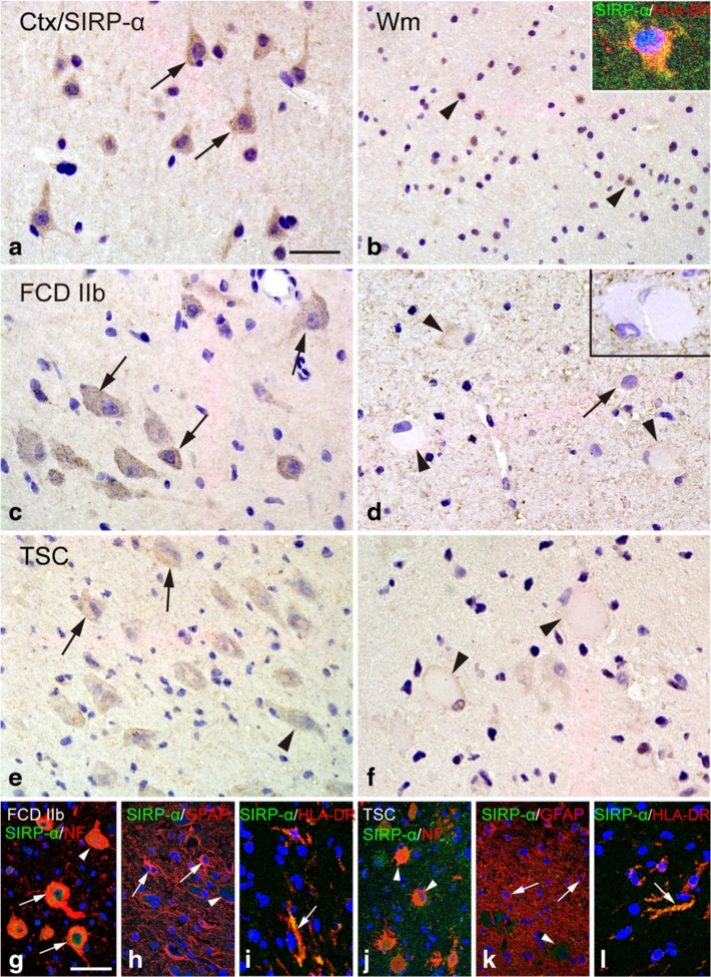
SIRP-α IR in control, FCD IIb, and TSC specimens. **a**, **b** SIRP-α IR in control specimens. **a** Strong somatic SIRP-α IR in neurons (*arrows*) within cortex. **b** Moderate SIRP-α IR in glial cells (*arrowheads*) within white matter and co-localization of SIRP-α (*green*) and HLA-DR (*red*) in a microglia (*inset*). **c**, **d** SIRP-α IR in cortical lesions of FCD IIb specimens. **c** Weak SIRP-α IR was occasionally detected in some dysmorphic neurons (*arrows*). **d** Negative balloon cells (*arrowheads*). **e**, **f** SIRP-α IR in cortical tubers of TSC specimens. **e** Weak SIRP-α IR in dysmorphic neurons (*arrows*) and a negative dysmorphic neurons (*arrowheads*). **f** Negative giant cells (*arrowheads*). **g**–**i** Double labeling in cortical lesions of FCD IIb specimens. **g** Co-localization of SIRP-α (*green*) with NF (*red*) in some dysmorphic neurons (*arrows*). **h** Absence of co-localization between SIRP-α (*green*) and GFAP (*red*) in astrocytes (*arrows*: astrocytes, *arrowhead*: dysmorphic neuron). **i** Co-localization of SIRP-α (*green*) with HLA-DR (*red*) in microglia (*arrow*). **j**–**l** Double labeling in cortical tubers of TSC specimens. **j** Absence of co-localization between SIRP-α (*green*) and NF (*red*) in giant cells (*arrowheads*). **k** Absence of co-localization between SIRP-α (*green*) and GFAP (*red*) in astrocytes (*arrows*: astrocytes, *arrowhead*: giant cell). **l** Co-localization of SIRP-α (*green*) with HLA-DR (*red*) in microglia (*arrow*). Scale bars: **a** 30 μm; **b**, **e** = 50 μm; **c** 35 μm; **d** 35 μm; **f** 30 μm; **g**, **h**, **j**, and **k** 50 μm; **i**, **l** 25 μm

In histologically normal autopsy specimens, SIRP-α strongly expressed in pyramidal neurons within cortex (Fig. 6a) and showed moderate IR in glial cells within white matter (Fig. 6b). FCD IIb and TSC specimens displayed weak or undetectable SIRP-α IR in dysmorphic neurons (Fig. 6c, e). Balloon cells of FCD IIb and giant cells of TSC did not exhibit detectable SIRP-α IR (Fig. 6d, f). The IR score of SIRP-α was dramatically lower in both the FCD IIb and TSC specimens than that in controls (*P* < 0.05; Fig. 4b) but showed no significant correlation with the number of HLA-DR-positive cells in FCD IIb (Fig. 5b) and TSC (Fig. 5e). Double labeling demonstrated the co-expression of SIRP-α IR with NF in some dysmorphic neurons and giant cells (Fig. 6g, j) and with HLA-DR in certain microglia (Fig. 6i, l), whereas the absence of SIRP-α IR was observed in GFAP-positive astrocytes (Fig. 6h, k).

### CD200

**Fig. 7:**
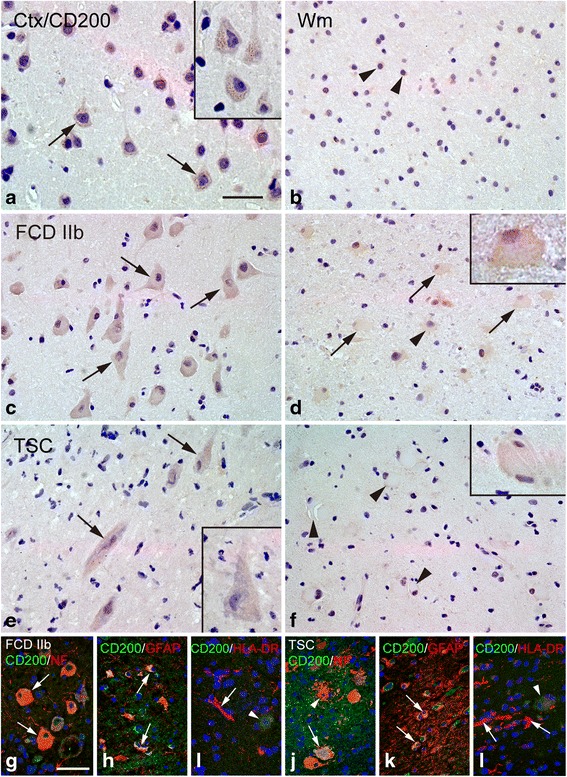
CD200 IR in control, FCD IIb, and TSC specimens. **a**, **b** CD200 IR in control specimens. **a** Moderate to strong somatic CD200 IR in neurons (*arrows* and *inset*) within cortex. **b** Weak CD200 IR in white matter (*arrowheads*). **c**, **d** CD200 IR in cortical lesions of FCD IIb specimens. **c** Weak CD200 IR in dysmorphic neurons (*arrows*). **d** Weak CD200 IR in balloon cells (*arrows*). **e**, **f** CD200 IR in cortical tubers of TSC specimens. **e** Weak CD200 IR in dysmorphic neurons (*arrows* and *inset*). **f** Weak CD200 IR in a giant cell (*inset*) and negative giant cells (*arrowheads*). **g**–**i** Double labeling in cortical lesions of FCD IIb specimens. **g** Co-localization of CD200 (*green*) with NF (*red*) in dysmorphic neurons (*arrows*). **h** Co-localization of CD200 (*green*) with GFAP (*red*) in reactive astrocytes (*arrows*: astrocytes, *arrowhead*: dysmorphic neuron). **i** Absence of co-localization between CD200 (*green*) with HLA-DR (*red*) in microglia (*arrow*: microglia, *arrowhead*: dysmorphic neuron). **j**–**l** Double labeling in cortical tubers of TSC specimens. **j** Co-localization of CD200 (*green*) and NF (*red*) in certain giant cell (*arrow*). **k** Co-localization of CD200 (*green*) with GFAP (*red*) in reactive astrocytes (*arrows*: astrocytes, *arrowhead*: giant cell). **l** Absence of co-localization between CD200 (*green*) and HLA-DR (*red*) in microglia (*arrows*: microglia, *arrowhead*: giant cell). *Scale bars*: **a** 30 μm; **b**–**l** 50 μm

In histologically normal autopsy specimens, moderate to strong CD200 IR was observed in pyramidal neurons within cortex (Fig. 7a). Weak and sporadic CD200 IR was detected within white matter (Fig. 7b). In FCD IIb and TSC specimens, weak CD200 IR was observed in dysmorphic neurons (Fig. 7c, e). Weak CD200 IR was also detected in balloon cells of FCD IIb (Fig. 7d). Giant cells of TSC exhibited weak or undetectable CD200 IR (Fig. 7f). The IR score of CD200 was significantly downregulated in both FCD IIb and TSC specimens versus the controls (*P* < 0.05; Fig. 4c) and showed significant negative correlation with the number of HLA-DR-positive cells in FCD IIb (Fig. 5c) and TSC (Fig. 5f). Double labeling showed that CD200 IR was co-localized with NF and GFAP in certain dysmorphic neurons, giant cells, and reactive astrocytes (Fig. 7g, h, j, and k). HLA-DR-positive microglia did not express CD200 (Fig. 7i, l).

### CD200R

**Fig. 8:**
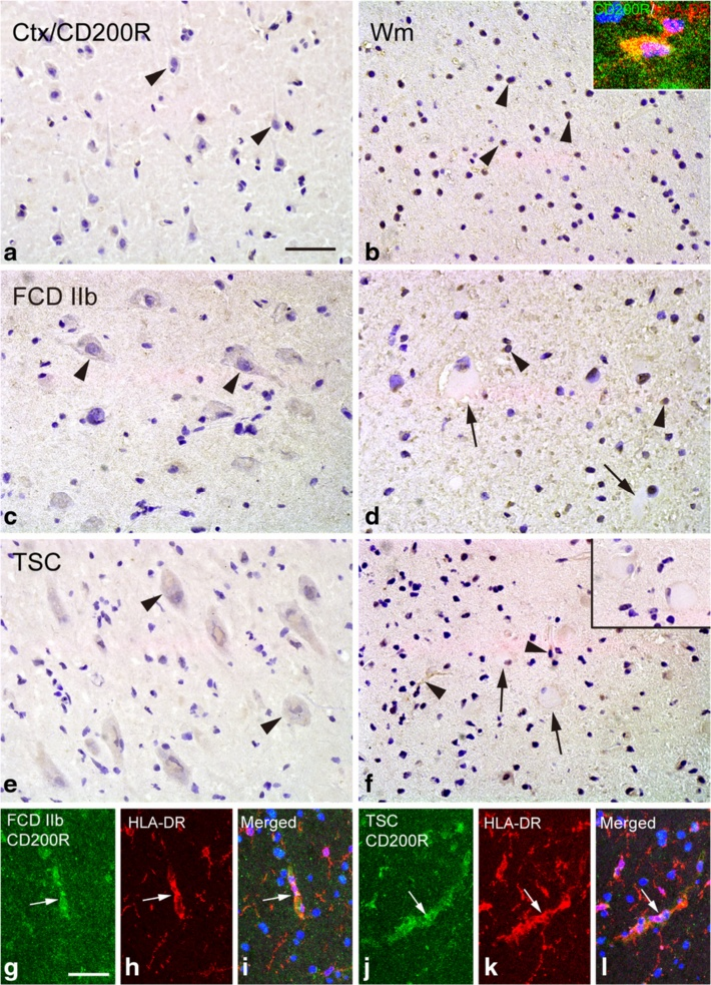
CD200R IR in control, FCD IIb, and TSC specimens. **a**, **b** CD200R IR in control specimens. **a** Undetectable CD200 IR in neurons (*arrows* and *inset*) within cortex. **b** Weak and sporadic CD200R IR in white matter (*arrowheads*) and co-localization of CD200R (*green*) and HLA-DR (*red*) in a microglia (*inset*). **c**, **d** CD200 IR in cortical lesions of FCD IIb specimens. **c** CD200R IR was not observed in dysmorphic neurons (*arrowheads*). **d** CD200R IR was not found in balloon cells (*arrows*) but was detected in certain glial cells (*arrowheads*). **e**, **f** CD200R IR in cortical tubers of TSC specimens. **e** Negative dysmorphic neurons (*arrowheads*). **f** Weak CD200R IR in glial cells (*arrowheads*) and negative giant cells (*arrows*). **g**–**i** Double labeling in cortical lesions of FCD IIb specimens shows co-localization of CD200R (*green*) with HLA-DR (*red*) in microglia (*arrow*). **j**–**l** Double labeling in cortical tubers of TSC specimens shows co-localization of CD200R (*green*) with HLA-DR (*red*) in microglia (*arrow*). *Scale bars*: **a**–**c**, **e**–**f** 50 μm; **d** 40 μm; **g**–**l** 35 μm

In histologically normal autopsy specimens, we did not detect CD200R IR in neurons within the cortex but observed weak and sporadic IR in glial cells within white matter (Fig. 8a, b). In FCD IIb and TSC specimens, CD200 IR was not observed in dysmorphic neurons (Fig. 8c, e), balloon cells (Fig. 8d), or giant cells (Fig. 8f) but was detected in certain glial cells with weak IR (Fig. 8d, f). The IR score of CD200R was not significantly changed in both the FCD IIb and TSC specimens compared with the controls (*P* > 0.05; Fig. 4d). Double labeling showed that CD200R IR was co-localized with HLA-DR in certain microglia (Fig. 8g–l).

### Effects of soluble human recombinant CD47 Fc and CD200 Fc on the production of IL-1β, IL-6, and IL-17

**Fig. 9:**
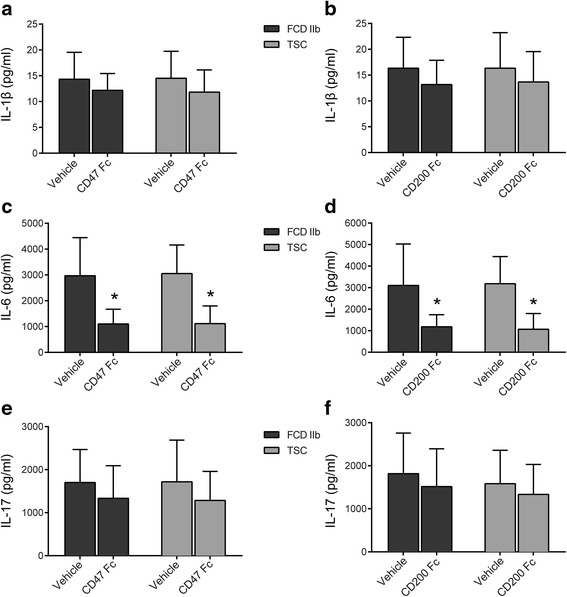
Effects of soluble recombinant human CD47 Fc Chimera protein and CD200 Fc chimera protein on the production of IL-1β, IL-6, and IL-17, as measured by ELISAs. IL-1β release was not inhibited by CD47 Fc (**a**) or CD200 Fc (**b**). Both CD47 Fc (**c**) and CD200 Fc (**d**) reduced the IL-6 production. The IL-17 release was not suppressed by CD47 Fc (**e**) or CD200 Fc (**f**). These data are representative of four experiments. The *bar charts* represent mean ± SD, Mann–Whitney *U* test, *P < 0.05

The concentration of cytokines was measured by ELISAs. Both CD47 Fc and CD200 Fc could reduce the IL-6 production (*P* < 0.05; Fig. 9c, d). Interestingly, IL-1β and IL-17 that were closely associated with IL-6 in epileptogenic lesions of MCD were not suppressed by CD47 Fc (*P* > 0.05; Fig. 9a, b) or CD200 Fc (*P* > 0.05; Fig. 9e, f).

### IL-4 mRNA expression and its correlation with CD200 mRNA level

**Fig. 10:**
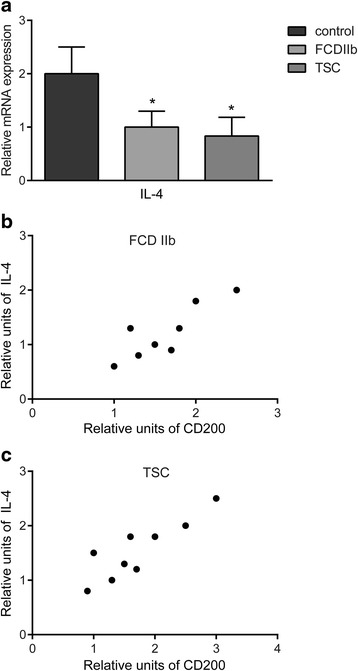
IL-4 mRNA expression in control, FCD IIb, and TSC specimens and the correlation between IL-4 and CD200 mRNA levels in FCD IIb and TSC specimens. **a** Quantitative real-time PCR analysis showed that IL-4 mRNA level was significantly decreased in FCD IIb and TSC compared with control specimens. There was no significant difference in IL-4 mRNA expression between FCD IIb and TSC specimens. The *bar charts* represent mean ± SD, *P < 0.05, FCD IIb, TSC versus controls, Kruskal–Wallis test, and Bonferroni correction. Scatter plot showing the significant positive correlation between IL-4 and CD200 mRNA levels in FCD IIb (**b**) (*P* < 0.05, *r* = 0.79) and TSC (**c**) (*P* < 0.01, *r* = 0.82). Correlation analysis performed with Spearman’s rank correlation coefficient

Previous studies showed that IL-4 and IL-13 increase the expression of CD200/CD200R [31, 32], so we investigated the mRNA expression of IL-4 and IL-13 in control, FCD IIb, and TSC specimens and the correlations with CD200 level. Quantitative real-time PCR analysis showed that IL-4 mRNA level was significantly decreased in FCD IIb and TSC compared with control specimens (*P* < 0.05; Fig. 10a). Scatter plot showing the significant positive correlation between IL-4 and CD200 mRNA levels in FCD IIb (*P* < 0.05, *r* = 0.79; Fig. 10b) and TSC (*P* < 0.01, *r* = 0.82; Fig. 10c). Consistent with a previous study [32], we fail to detect IL-13 expression in our study (data not shown).

## Discussion

In the present study, we demonstrate that the expression of the immune inhibitory molecules CD47 and its receptor, SIRP-α, and CD200 are downregulated in surgically resected brain tissues from patients with FCD IIb and TSC, both of which are associated with medically intractable pediatric epilepsy, whereas the expression of CD200 receptor, CD200R, is not significantly altered. In addition, we show that both soluble human recombinant CD47 Fc and CD200 Fc could reduce IL-6 release in the living epileptogenic brain slices FCD IIb and TSC patients in vitro. These findings provide evidence of an immune inhibitory deficit involving CD47/SIRP-α and CD200/CD200R pathways in human epileptogenic lesions of FCD IIb and TSC patients.

CD47 is ubiquitously expressed in various resident cells of the CNS, transducing an inhibitory signal via its receptor, SIRP-α, which is present on microglia/macrophages and neurons. It was reported that CD47 expression is decreased in human brain lesions of multiple sclerosis (MS), a CNS autoimmune neuroinflammatory disease, whereas SIRP-α expression is unchanged, suggesting that decreased CD47 expression contribute to a disturbed equilibrium in macrophage and microglia activation in MS lesions and may result in a proinflammatory predisposition in the area surrounding chronic active lesions [44]. Han et al. further demonstrated that CD47 is expressed in normal myelin and in foamy macrophages and reactive astrocytes within active MS lesions (not in reactive astrocytes within chronic MS lesions) and displays Janus-like opposing effects on MS pathogenesis by interacting with SIRP-α, which is likely caused by the expression of CD47 in different cell types and locations [45].

Our results showed that the expression of CD47 was downregulated at both the messenger RNA and protein levels in epileptogenic lesions of FCD IIb and TSC and that the SIRP-α expression was also decreased, which is different from what has been observed in human MS. In the control specimens, CD47 and SIRP-α were relatively highly expressed in neurons but expressed at lower levels in dysmorphic neurons, balloon cells, and giant cells in epileptogenic lesions of FCD IIb and TSC patients. In addition, weak to moderate CD47 and SIRP-α IR were detected in certain microglia in both control and epileptogenic lesions, which is in agreement with previous studies showing that both CD47 and SIRP-α are expressed in cultured microglia [19, 22]. Moreover, significant negative correlation between the IR score of CD47 and the number of HLA-DR-positive cells, representing activated microglia [41], was observed in FCD IIb and TSC specimens. These findings indicate an inefficient combination of CD47 and SIRP-α not only in neuron-neuron interactions but also in neuron-microglia interactions. On the one hand, neuronal CD47/SIRP-α complex has been demonstrated to play important roles in neuronal network formation, as evident by the observation that CD47/SIRP-α is able to promote neurite and dendritic spine formation in cultured hippocampal neurons [46, 47]. Thus, CD47-SIRP-α deficiency in the misshapen cells (e.g., dysmorphic neurons, balloon cells, giant cells) within the epileptogenic lesions might contribute to the abnormal neuronal migration and differentiation during brain development in FCD IIb and TSC patients [48, 49]. On the other hand, since the interaction between neuronal CD47 and microglial SIRP-α results in the inhibition of microglia [50], the inefficient CD47/SIRP-α interaction between misshapen cells and microglia within the epileptogenic lesions may conduce to microglial activation, which abundantly occurs in epileptogenic lesions of FCD IIb and TSC and is thought to perpetuate and prolong the inflammation [41, 51–53]. However, the specific roles of CD47/SIRP-α in these processes of FCD IIb and TSC require further investigation. Although CD47 and SIRP-α were found to be expressed in microglia in both control and epileptogenic lesions of FCD IIb and TSC, the role of CD47/SIRP-α interaction in microglia is still under investigation [22].

Similar to CD47/SIRP-α, CD200/CD200R is another anti-inflammatory system in the brain. CD200 is broadly expressed on neurons and other cells and mediates inhibitory signals via its receptor, CD200R, on cells of the myeloid lineage, including macrophage/microglia. A previous study demonstrated that CD200-deficient mice exhibit an increased number of microglia/macrophages with a more activated phenotype, as well as an accelerated and aggravated course of EAE [28]. Conversely, mice with elevated neuronal expression of CD200 due to a mutation in the Wlds gene display an attenuated EAE course with decreased CNS macrophage/microglial accumulation [54]. Moreover, the level of CD200, together with its receptor, CD200R, has been reported to be decreased in Alzheimer’s disease (AD), a neurodegenerative disease, that is characterized by ongoing chronic inflammation in the brain lesions [32, 55].

In the present study, we provided the first evidence of cell-specific downregulation of both mRNA and protein levels of CD200 in epileptogenic lesions of FCD IIb and TSC patients, while the expression of its receptor, CD200R, was not significantly changed. Accordingly, similar alterations in its expression have been reported in human MS brain lesions [44]. Immunohistochemical analysis showed that CD200 was consistently and predominantly expressed in neurons within histologically normal cortex, while its expression was dramatically decreased in misshapen cells in epileptogenic lesions of FCD IIb and TSC patients, including dysmorphic neurons, balloon cells, and giant cells. We did not observe CD200R expression in neurons of the control specimens or in misshapen cells within epileptogenic tissues, but we detected CD200R expression in some microglia in both control specimens and epileptogenic lesions of FCD IIb and TSC patients. Additionally, CD200 IR score displayed significant negative correlation with the number of HLA-DR-positive cells (activated microglia) in FCD IIb and TSC specimens. It is hypothesized that the decreased expression of CD200 in these misshapen cells may lead to the inefficient interaction between CD200 and CD200R, which subsequently contributes to microglial activation and the concomitant chronic inflammation in epileptogenic lesions of FCD IIb and TSC patients.

In addition to its localization to neurons, CD200 expression has also been observed in reactive astrocytes within brain lesions of human MS and AD patients [32, 56]. In agreement, we also detected weak and sporadic expression of CD200 in reactive astrocytes that were abundantly present in the epileptogenic lesions of MCD [57]. This suggests that CD200-mediated immune suppression might also occur through astrocyte-microglia interactions during the inflammatory epileptogenic lesions of FCD IIb and TSC. However, the evaluation of CD200 function in reactive astrocytes needs to be further investigated.

Evidence has recently been presented showing inflammatory response markers in developing cortical tubers of fetal TSC brain ranging from 23 to 38 gestational weeks, including major histocompatability complexes classes I and II, Toll-like receptors 2 and 4, and receptor for advanced glycation end products [58]. These findings are relevant and significant in demonstrating that at least some inflammation in tuberous sclerosis begin very early and thus may be an integral part of pathogenesis, not just a reactive change to it.

Previous studies have revealed that many proinflammatory cytokines are upregulated in epileptogenic lesions and play pivotal roles in the epileptogenesis of FCD IIb and TSC patients, including IL1-β, IL-6, and IL-17 [15–17, 30]. It has been demonstrated that CD47 Fc could decrease proinflammatory cytokine release, including IL-6, IL-12, tumor necrosis factor α, and interferon-γ, by binding to its receptor, SIRP-α, in human dendritic cells [24]. CD200 Fc has also been reported to suppress IL-6 and IL1-β production by engaging CD200R in activated microglia [36].

In our in vitro assay, we showed that soluble human recombinant CD47 Fc and CD200 Fc could reduce IL-6 release but did not suppress IL1-β or IL-17 production, in living cortical brain slices from patients with FCD IIb and TSC. Therefore, we speculate that CD47 and CD200 exert an anti-inflammatory function in epileptogenic lesions of FCD IIb and TSC by suppressing the production of proinflammatory cytokines, such as IL-6, but the mechanism requires further exploration. Due to the limited amount of human samples, we did not examine the effect of CD47 Fc and CD200 Fc on the inhibition of these proinflammatory cytokines at different doses and time points.

It has been demonstrated that IL-4 markedly increases CD200 expression in cultured hippocampal neurons and that CD200 staining was significantly decreased in neurons prepared from IL-4 knockout mice compared with wild-type ones [31]. IL-4 has also been shown to upregulate CD200R expression in human microglia and microphages [32]. In this study, we showed that IL-4 mRNA level was significantly decreased in FCD IIb and TSC specimens compared with the control samples and displayed positive correlation with CD200 mRNA level. Since IL-4 is a well-recognized anti-inflammatory cytokine [59], our findings are suggestive that in epileptogenic lesions of FCD IIb and TSC, anti-inflammatory cytokines like IL-4 are deficient and that decreased expression of CD200 may be partially caused by insufficient IL-4 level.

## Conclusions

In this study, we have described reduced expression of CD47/SIRP-α and CD200 in epileptogenic brain tissues from patients with FCD IIb and TSC, while CD200R expression is unchanged. Our results suggest that microglial activation may be partially caused by CD47/SIRP-α- and CD200/CD200R-mediated reductions in the immune inhibitory pathways within FCD IIb and TSC cortical lesions where chronic neuroinflammation has been established. Upregulation or activation of CD47/SIRP-α and CD200/CD200R has been demonstrated to be beneficial in several immune inflammatory disease models. We speculate that increasing CD47/SIRP-α and CD200/CD200R expression may also have protective roles in FCD IIb and TSC, since we have revealed that CD47 Fc and CD200 Fc could reduce IL-6 release, a key proinflammatory cytokine involved in FCD IIb and TSC. However, due to the limitation of studies using human samples and the absence of genuine models of FCD IIb and TSC [60], the evidence we provide here is largely descriptive. More functional studies are needed to explore the inherent role of CD47/SIRP-α and CD200/CD200R pathways in epileptogenesis of MCD, as well as possible therapeutic interventions based on the regulation of the these two systems.

## Additional file

Additional file 1: Figure S1. Hematoxylin/eosin staining of normal-appearing cortical area and the dysplastic area of TSC and FCD IIb. (**a**) Normal-appearing cortical area. (**b**) Dysplastic area of FCD IIb, dysmorphic neurons (arrows) and balloon cells (arrowheads). (**c**) Dysplastic area of TSC, dysmorphic neurons (arrows) and giant cells (arrowheads). Scale bars: 100 μm for all panels. (TIF 4.12 mb)

## Abbreviations

ELISAs: enzyme-linked immune sorbent assays
EAE: experimental autoimmune encephalomyelitis
FCD IIb: focal cortical dysplasia type IIb
GFAP: glial fibrillary acidic protein
HLA-DR: human leukocyte antigen-DR
MCD: malformations of cortical development
MS: multiple sclerosis
NF: neurofilament
qPCR: quantitative real-time polymerase chain reaction
SIRP-α: signals regulatory protein α
TSC: tuberous sclerosis complex
