# Supplementary figures and images for: Downregulation of CD47 and CD200 in patients with focal cortical dysplasia type IIb and tuberous sclerosis complex
Source: J Neuroinflammation. 2016 Apr 19;13:85. doi: 10.1186/s12974-016-0546-2 (PMC4837553; doi:10.1186/s12974-016-0546-2)

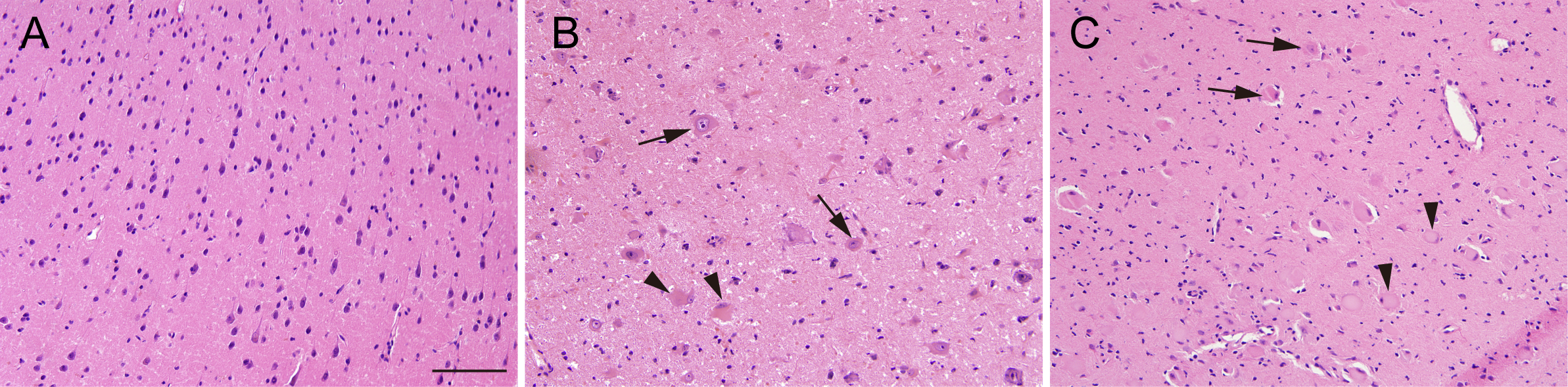

Supplement: Additional file 1: Figure S1. — Hematoxylin/eosin staining of normal-appearing cortical area and the dysplastic area of TSC and FCD IIb. (a) Normal-appearing cortical area. (b) Dysplastic area of FCD IIb, dysmorphic neurons (arrows) and balloon cells (arrowheads). (c) Dysplastic area of TSC, dysmorphic neurons (arrows) and giant cells (arrowheads). Scale bars: 100 μm for all panels. (TIF 4.12 mb) [file 12974_2016_546_MOESM1_ESM.tif]
